# Geographic Clusters of Primary Biliary Cirrhosis

**DOI:** 10.1080/10446670310001626526

**Published:** 2003

**Authors:** Saif Abu-Mouch, Carlo Selmi, Gordon D. Benson, Thomas P. Kenny, Pietro Invernizzi, Massimo Zuin, Mauro Podda, Lorenzo Rossaro, M. Eric Gershwin

**Affiliations:** 1 Hepatology Clinic Hillel Yaffe Medical Center Hadera Israel; 2 Division of Rheumatology Allergy and Clinical Immunology University of California at Davis School of Medicine Davis CA USA; 3 Division of Internal Medicine Department of Medicine, Surgery and Dentistry San Paolo School of Medicine University of Milan Italy; 4 Robert Wood Johnson Medical School University of Medicine and Dentistry of New Jersey Camden NJ USA; 5 Division of Transplant Medicine University of California at Davis School of Medicine Sacramento CA USA

## Abstract

Genetic and environmental factors have been widely suggested to contribute to the pathogenesis of primary biliary cirrhosis (PBC), an autoimmune disease of unknown etiology leading to destruction of small bile ducts. Interestingly, epidemiologic data indicate a variable prevalence of the disease in different geographical areas. The study of clusters of PBC may provide clues as to possible triggers in the induction of immunopathology. We report herein four such unique PBC clusters that suggest the presence of both genetic and environmental factors in the induction of PBC. The first cluster is represented by a family of ten siblings of Palestinian origin that have an extraordinary frequency of PBC (with 5/8 sisters having the disease). Second, we describe the cases of a husband and wife, both having PBC. A family in which PBC was diagnosed in two genetically unrelated individuals, who lived in the same household, represents the third cluster. Fourth, we report a high prevalence of PBC cases in a very small area in Alaska. Although these data are anedoctal, the study of a large number of such clusters may provide a tool to estimate the roles of genetics and environment in the induction of autoimmunity.


*Clinical & Developmental Immunology*, June–December 2003 Vol. 10 (2–4), pp. 127–131

Taylor & Francis healthsciences

### Geographic Clusters of Primary Biliary Cirrhosis

SAIF ABU-MOUCH[a,*], CARLO SELMI[b,c,*], GORDON D. BENSON[d], THOMAS P. KENNY[b], PIETRO INVERNIZZI[c], MASSIMO ZUIN[c], MAURO PODDA[c], LORENZO ROSSARO[e] and M. ERIC GERSHWIN[b,†]

[a]*Hepatology Clinic, Hillel Yaffe Medical Center, Hadera, Israel;* [b]*Division of Rheumatology, Allergy and Clinical Immunology, University of California at Davis, School of Medicine,Davis, CA, USA;* [c]*Division of Internal Medicine, Department of Medicine, Surgery and Dentistry, San Paolo School of Medicine, University of Milan, Italy;* [d]*Robert Wood Johnson Medical School, University of Medicine and Dentistry of New Jersey, Camden, NJ, USA;* [e]*Division of Transplant Medicine, University of California at Davis, School of Medicine, Sacramento, CA, USA*

Genetic and environmental factors have been widely suggested to contribute to the pathogenesis of primary biliary cirrhosis (PBC), an autoimmune disease of unknown etiology leading to destruction of small bile ducts. Interestingly, epidemiologic data indicate a variable prevalence of the disease in different geographical areas. The study of clusters of PBC may provide clues as to possible triggers in the induction of immunopathology. We report herein four such unique PBC clusters that suggest the presence of both genetic and environmental factors in the induction of PBC. The first cluster is represented by a family of ten siblings of Palestinian origin that have an extraordinary frequency of PBC (with 5/8 sisters having the disease). Second, we describe the cases of a husband and wife, both having PBC. A family in which PBC was diagnosed in two genetically unrelated individuals, who lived in the same household, represents the third cluster. Fourth, we report a high prevalence of PBC cases in a very small area in Alaska. Although these data are anedoctal, the study of a large number of such clusters may provide a tool to estimate the roles of genetics and environment in the induction of autoimmunity.

*Keywords*: Autoimmunity; Environment; Genetics; Geoepidemiology

### INTRODUCTION

Primary biliary cirrhosis (PBC) is an autoimmune disease characterized by a female preponderance (9:1 female to male ratio) with most cases occurring between 40 and 60 years of age (Kaplan, 1996). Autoantibodies to mitochondrial antigens (AMA) are detectable in 85–95% of cases of PBC and often occur long before clinical signs or symptoms appear; this test appears to be highly specific for PBC (Kaplan, 1996). The autoantigens reactive against AMA have been identified as subunits of a functionally related family of enzymes, the 2-oxo-acid dehydrogenases, and particularly as pyruvate dehydrogenase E2 (PDC-E2), oxoglutaric dehydrogenase E2 (OGDC-E2), and branched-chain a-ketoacid dehydrogenase E2 (BCOADC-E2) (Gershwin *et al.*, 2000). Liver histology in patients with PBC shows progressive destruction of small intrahepatic bile ducts, and ultimately cirrhosis; four histological stages are described accordingly, spanning from mild inflammatory infiltrate to frank liver cirrhosis (Ludwig *et al.*, 1978).

The genetic background appears important in determining the susceptibility and possibly the severity of PBC (Tanaka *et al.*, 2001). In the case of major histocompatibility complex (MHC) variants, for example, the associations demonstrated for other autoimmune diseases have not been confirmed in PBC or appear to be limited to certain geographical areas (Donaldson *et al.*, 1994; Agarwal *et al.*, 1999; Tanaka *et al.*, 2001). Beside genetic predisposition, a number of environmental factors, including molecular mimicry by either microorganisms or xenobiotics have also been proposed (Long *et al.*, 2001; Van de Water *et al.*, 2001; Long *et al.*, 2002; Palmer *et al.*, 2002). One resulting hypothesis is that environmental factors may trigger PBC in genetically predisposed individuals. Several studies, in fact, indicate that family members of known cases with PBC present a significantly higher risk of developing the disease (Tsuji *et al.*, 1999). Population-based studies attempting to estimate the prevalence and incidence of PBC have introduced the concept of geoepidemiology of the disease, with higher prevalence in England and Sweden, although a number of biases could not be ruled out (Parikh-Patel *et al.*, 1999). Available evidence estimating the prevalence and incidence of PBC in different geographical areas is shown in Table I. The study of clusters of well-defined PBC cases may provide a helpful tool to estimate the relative roles of

*These authors contributed equally to this work.
†Corresponding author. Tel.: +1-530-752-2884. Fax: +1-530-752-4669. E-mail: megershwin@ucdavis.edu

ISSN 1740-2522 print/ISSN 1740-2530 online © 2003 Taylor & Francis Ltd
DOI: 10.1080/10446670310001626526

TABLE I Synopsis of population-based epidemiological studies of PBC (modified and updated from Parikh-Patel *et al.* (1999)

| Year | Location | No. of cases | Case finding methods | Diagnostic criteria | Incidence (per million, per year) | Prevalence (per million) | Gender ratio (M/F) |
|---|---|---|---|---|---|---|---|
| 1980 | Sheffield, UK | 34 | PS, lab reports | AMA+ and LFTs or liver histology | 5.8 | 54 | 1:16 |
| 1980 | Dundee, UK | 21 | Liver histology | AMA+ and liver histology | 10.6 | 40.2 | 1:9.5 |
| 1983 | Newcastle, UK | 117 | Hospital registers, lab reports, death certificates | AMA+, LFTs, and liver histology | 10 | 37–144 | 1:14 |
| 1984 | Malmoe, Sweden | 33 | PS, lab reports, death certificates | AMA+, LFTs, and liver histology | 4–24 | 28–92 | 1:3 |
| 1984 | Western Europe | 569 | PS | Non uniform | 4 | 23 (5–75) | 1:10 |
| 1985 | Orebro, Sweden | 18 | Lab reports | AMA+, LFTs, and liver histology | 14 | 128 | 1:3.5 |
| 1987 | Glasgow, UK | 373 | Lab reports | AMA+, liver histology | 11–15 | 70–93 | – |
| 1990 | Umea, Sweden | 111 | PS, hospital registers, lab reports | Liver histology | 13.3 | 151 | 1:6 |
| 1990 | Ontario, Canada | 225 | PS | AMA+, liver histology | 3.26 | 22.4 | 1:13 |
| 1990 | Northern England | 347 | PS, hospital admission data, lab reports | AMA+ and LFTs or liver histology | 19 | 129–154 | 1:9 |
| 1995 | Victoria, Australia | 84 | PS, hospital records | AMA+, LFTs, and liver histology | – | 19.1 | 1:11 |
| 1995 | Estonia | 69 | PS, hospital admission data, lab reports | AMA+ and LFTs and liver histology | 2.27 | 26.9 | 1:22 |
| 1997 | Newcastle, UK | 160 | PS, hospital admission data, lab reports, death certificates | AMA+, LFTs, and liver histology | 14–32 | 240 | 1:10 |
| 2000 | Olmsted county, MN (USA) | 46 | Hospital records | LFTs, and AMA+ or liver histology | 27 | 402 | 1:8 |

Abbreviations used: PS physician survey; AMA anti-mitochondrial antibodies; LFTs liver function tests genetics and environment in the induction of PBC. The clusters described herein are examples of how these two factors can be studied.

### MATERIALS AND METHODS

### Definition of PBC

The diagnosis of PBC was in all cases performed according to internationally accepted criteria (Kaplan, 1996). Briefly, two out of three conditions (elevated serum alkaline phoshatase for longer than 6 months, positivity for anti-mitochondrial antibodies, or diagnosis at histology) had to be fulfilled to confirm the diagnosis of PBC.

### Determination of AMA Reactivity

AMA determination was performed using recombinant mitochondrial proteins as previously described (Miyakawa *et al.*, 2001). Sera presenting reactivity at titre higher than 1:80 against one or more of the recombinant proteins (PDC-E2, BCOADC-E2, OGDC-E2) were considered to be AMA positive.

### Clusters of PBC

The characteristics of the subjects included in the four clusters are summarized in Table II.

### *Cluster 1*

A family of ten siblings (age range 27–49 years) of Palestinian origin currently living in the same area have an extraordinary high prevalence of PBC. We collected blood samples from all 8 sisters and 2 brothers, as well as from the mother, and tested sera for AMA, using recombinant mitochondrial antigens as previously described (Miyakawa *et al.*, 2001). Five sisters (age range 27–47 years) presented a confirmed clinical diagnosis of PBC within a 10-year interval (1992–2002), including the presence of high titre AMA with similar patterns of reactivity and clinical stages of disease. Two other sisters had weak AMA reactivity, but no other evidence of PBC. Only one sister, both brothers, as well as the mother had neither detectable AMA reactivity nor signs of PBC. Eight different commercially available polymorphic microsatellite markers (Map Pairs, Res Gen, Invitrogen corp, Carlsbad, CA) were tested to study the parentage of this family. Six markers are located on six different chromosomes (D4S1647, D5S815, D7S796, D10S1146, D21S1910, D22S683), while 2 are found on chromosome 6 close to the HLA loci (D6S265, D6S299). Genotypes were determined using PCR amplification of 10 ng of genomic DNA in 25 μl total volume reactions. PCR solution included 10 mM dNTP mix, 25 mM $MgCl_2$ solution, 10 × PCR Gold Buffer, 1 Unit of AmpliTaq GoldTM polymerase

TABLE II Characteristics of the subjects included in the four PBC clusters

| Cluster | Subject | Year of birth | AMA | PBC (year of diagnosis) |
|---|---|---|---|---|
| 1 | Mother | 1937 | NEG | − |
| | 1st daughter | 1954 | POS | − |
| | 2nd daughter | 1956 | POS | + (1992) |
| | 3rd daughter | 1960 | POS | + (1996) |
| | 4th daughter | 1963 | POS | + (1997) |
| | 5th daughter | 1965 | POS | + (2002) |
| | 6th daughter | 1968 | NEG | − |
| | 7th daughter | 1973 | POS | − |
| | 8th daughter | 1976 | POS | + (2001) |
| | 1st son | 1958 | NEG | − |
| | 2nd son | 1970 | NEG | − |
| 2 | Wife | 1947 | POS | + (1990) |
| | Husband | 1952 | POS | + (1995) |
| 3 | Grandmother | 1911 | POS | + (1977) |
| | Daughter | 1931 | POS | − |
| | Daughter in-law | 1931 | POS | + (1987) |
| | Granddaughter | 1964 | POS | − |
| 4 | Patient #1 | 1940 | POS | + (2000) |
| | Patient #2 | 1935 | POS | + (1998) |
| | Patient #3 | 1956 | POS | + (2001) |
| | Patient #4 | 1935 | POS | + (2000) |
| | Patient #5 | 1932 | ? | + (1973) |
| | Patient #6 | 1951 | POS | + (1992) |

(Applied Biosystems, Foster City, CA, USA), 20 uM of each primer, and DNAase-RNAase-free water up to a 25 μl volume. Forward primers were labeled with ATP(y-33P) (Perkin Elmer, Boston, MA, USA) using a T4 Polynucleotide Kinase system (Invitrogen). Amplification was carried out using Programmable Thermal Controller (MJ Research Inc., Walthamm MA, USA) under the following conditions: 10′ denaturation at 94°C; 9 cycles of 45″ at 94°C, 45″ at 58°C to 50°C (−1°C per cycle), 60″ at 72°C; 35 cycles of 45″ at 94°C, 45″ at 50°C, 60″ at 72°C, followed by 7′ at 72°C. Upon completion of PCR, 1 μl of product was taken and added to 15 μl of dye and water and heated for 5 min at 95°C. Finally, 3 μl of product/dye solution were run onto a 7% polyacrylamide Bio-Rad Sequencing Gel (Bio-Rad Laboratories, Inc., Hercules, CA, USA) for 3 h at 60 W and then transferred to a Molecular Dynamics Phosphor Screen (Amersham Biosciences, Piscataway, NJ, USA). Gel results were analyzed using ImageQuaNT by Molecular Dynamics (Amersham Biosciences). The patterns obtained from the 10 siblings were fully compatible with a complete brotherhood (Pena and Chakraborty, 1994).

### *Cluster 2*

A case of husband and wife, both with a well-characterized diagnosis of PBC, live in the Northwestern region of the US. Disease developed after their marriage with diagnosis five years apart from each other (both individuals were 43 at the time of diagnosis). Despite being five years younger and being diagnosed five years after his wife, the male patient currently presents a histologically-proven more advanced disease. Interestingly, they were born in the same street of the same large city in the state of Michigan, without knowing each other for decades, and that the only recognizable possible risk factor for both individuals was previous cigarette smoking. Other proposed risk factors for PBC were also investigated and, interestingly, recurrent urinary tract infections were reported only by the female subject.

### *Cluster 3*

We describe a family from the state of New York in which the grandmother (our index case) had the diagnosis of PBC established in 1977. Her daughter, who remains asymptomatic, has shown a similar AMA pattern with a rising titer over 13 years. Interestingly, however, and suggesting a role for environmental factors in this cluster, we report the development of PBC in the daughter-in-law of our index case; this woman emigrated to the US from Korea and lived in the same household for two years and in the same neighborhood for over thirty years before being also diagnosed with PBC. Her daughter (granddaughter of our index case) was found to be repeatedly positive for AMA (with titres increasing over the past 14 years) without showing any other evidence of PBC. Clinically, the two defined cases present very different degrees of disease severity.

### *Cluster 4*

We describe a cluster of women with PBC in an area including two small cities in the state of Alaska (female population 4.588 in 2000). Between 1973 and 2001, 6 cases of PBC were diagnosed according to internationally accepted criteria among people currently or previously living for at least 10 years in that area.

Three of these cases, interestingly, had been working together as phone operators for several years before the diagnosis was made.

In order to estimate the prevalence of PBC in such area, we considered only the cases who were diagnosed during their residence in the area and still alive in 2000. Four cases (age range 44–65 years) fulfilled the criteria and all presented signs of early disease (histological stages I–II according to Ludwig *et al*[3]); the ethnicity was Caucasian in three cases and Native American in one patient. While the available data show that the prevalence of PBC is the US is expected to be 402/million (Kim *et al.*, 2000), the frequency among women population in the Alaskan area in 2000 can be estimated to be 870 per million.

### DISCUSSION

Factors conferring susceptibility to PBC or triggering autoimmunity remain poorly understood. Epidemiological data seem conflicting in determining the importance of genetic versus environmental factors. In fact, while PBC prevalence seem to present a geographical distribution (Table I), thus suggesting a strong role for exogenous factors (Parikh-Patel *et al.*, 1999), other data indicate a risk of developing the disease for a first-degree relative of an affected subject much higher compared to other autoimmune diseases, thus stressing a genetic susceptibility (Tsuji *et al.*, 1999). Beside this latter observation, relatives of affected individuals seem to develop the disease within a short time from the first case, thus possibly indicating again some environmental influence (Tsuji *et al.*, 1999). Many studies have concentrated on genetic factors in patients with PBC (Tanaka *et al.*, 2001) while most evidence about other factors comes from experimental studies (Long *et al.*, 2001; Leung *et al.*, 2003; Xu *et al.*, 2003).

A discrete number of genetic variants have been associated with the disease, although no definitive conclusion was reached. The strong association with HLA molecules observed in other autoimmune diseases has been not found in PBC and data about this aspect are either conflicting or apparently geographically limited (Tanaka *et al.*, 2001). Other polymorphisms have been indicated as able to confer susceptibility or to influence the progression of the disease but also in these cases, results are generally debated, conflicting, or non conclusive (Donaldson *et al.*, 1994; Agarwal *et al.*, 1999; Tanaka *et al.*, 2001).

Most data indicating an environmental influence in determining PBC have been collected for microorganisms and xenobiotics. Several lines of evidence support a role for infectious agents, especially bacteria and retroviruses, in the pathogenesis of PBC (van de Water *et al.*, 2001; Xu *et al.*, 2003). The microbial mechanism termed "molecular mimicry" is a hypothesis forwarded to account for breaking tolerance against mitochondrial antigens (van de Water *et al.*, 2001).

Xenobiotics are foreign compounds that may either alter or complex to defined self proteins, inducing a change in the molecular structure of the native protein sufficient to induce an immune response. Such immune responses may then result in the recognition of not only the modified protein, but also the native form. The chronic presence of the self-protein may thereafter perpetuate the immune response initiated by the xenobiotic-induced adduct thus leading to chronic autoimmunity. Beside the proposed role for microorganisms, experimental data have also shown a possible a role for some halogenated compounds in the induction of AMA and possibly of PBC (Long *et al.*, 2001; Leung *et al.*, 2003).

It is interesting to note familiar or environmental clusters of PBC cases which could, in a similar fashion as non-population based twin studies (comparing concordance rates among monozygotic and dizygotic twins), provide an estimate of the role of individual and exogenous factors in determining susceptibility to multifactorial diseases such as PBC. The only case of non-familiar cluster of PBC reported so far described an individual nursing a patient with PBC and later developing the disease (Douglas and Finlayson, 1979). We therefore report herein four interesting clusters of patients with PBC, although contrasting in their possible implications. It is interesting to note that in cluster #1 the mother of the offspring does not have any sign of PBC, indicating either that responsible genes could be of paternal origin or the presence of some environmental trigger that appeared or became active only in the later generation. In a similar fashion, cluster #3 cannot be explained only by genetic influence, because of the case occurred in the woman of Korean origin. It is worth to notice, moreover, that unpublished data seem to indicate a very low prevalence of PBC in the Korean region (Dr Hee-Sik Sun, Catholic University in Seoul, personal communication). Clusters #2 and #4, on the other hand, clearly indicate a role for non-individual factors, possibly represented by the household (cluster #2) or by the geographical area (cluster #4), as suggested in the past by some epidemiological data from English studies (Triger, 1980; Prince *et al.*, 2001). In three of the clusters described herein, a role for genetics seems unlikely because of the lack of consanguinity between the spouses (cluster #2) or the two women with PBC in cluster #3, and the different ethnic groups among the Alaskan patients (cluster #4). In cluster #2, information obtained about proposed risk factors for PBC (Parikh-Patel *et al.*, 2001) showed how in this particular case, history of recurrent urinary tract infections was not found in both individuals. Some unknown risk factors related with the professional history might be present in half of the cases in the Alaskan area. However, while the prevalence of PBC in this region has never been investigated, it should be noted that the estimated prevalence for PBC in the region of cluster #4 was found to be the highest ever reported for the disease (Parikh-Patel *et al.*, 1999). Finally, we note that these cases suggest once again that PBC shares key genetic and environmental factors, as well as suggesting a sort of 'multi-hit' pathogenesis (Donaldson *et al.*, 2001). The study of a significant number of such clusters may provide a tool to assess the relative roles of genetics and environment in the induction of PBC.

### References

Agarwal, K., Jones, D.E. and Bassendine, M.F. (1999) "Genetic susceptibility to primary biliary cirrhosis", *Eur. J. Gastroenterol Hepatol.* **11**, 603–606.

Donaldson, P., Doherty, D., Underhill, J. and Williams, R. (1994) "The molecular genetics of autoimmune liver disease", *Hepatology* **20**, 225–239.

Donaldson, P., Agarwal, K., Craggs, A., Craig, W., James, O. and Jones, D. (2001) "HLA and interleukin 1 gene polymorphisms in primary biliary cirrhosis: associations with disease progression and disease susceptibility", *Gut* **48**, 397–402.

Douglas, J.G. and Finlayson, N.D. (1979) "Are increased individual susceptibility and environmental factors both necessary for the development of primary biliary cirrhosis?", *Br. Med. J.* **2**, 419–420.

Gershwin, M.E., Ansari, A.A., Mackay, I.R., *et al.* (2000) "Primary biliary cirrhosis: an orchestrated immune response against epithelial cells", *Immunol. Rev.* **174**, 210–225.

Kaplan, M.M. (1996) "Primary biliary cirrhosis", *N. Engl. J. Med.* **335**, 1570–1580.

Kim, W.R., Lindor, K.D., Locke, G.R., 3rd, *et al.* (2000) "Epidemiology and natural history of primary biliary cirrhosis in a US community", *Gastroenterology* **119**, 1631–1636.

Leung, P.S., Quan, C., Park, O., *et al.* (2003) "Immunization with a xenobiotic 6-bromohexanoate bovine serum albumin conjugate induces antimitochondrial antibodies", *J. Immunol.* **170**, 5326–5332.

Long, S.A., Quan, C., van de Water, J., *et al.* (2001) "Immunoreactivity of organic mimeotopes of the E2 component of pyruvate dehydrogenase: connecting xenobiotics with primary biliary cirrhosis", *J. Immunol.* **167**, 2956–2963.

Long, S.A., van de Water, J. and Gershwin, M.E. (2002) "Antimitochondrial antibodies in primary biliary cirrhosis: the role of xenobiotics", *Autoimmun. Rev.* **1**, 37–42.

Ludwig, J., Dickson, E.R. and McDonald, G.S. (1978) "Staging of chronic nonsuppurative destructive cholangitis (syndrome of primary biliary cirrhosis)", *Virchows Arch. A Pathol. Anat. Histol.* **379**, 103–112.

Miyakawa, H., Tanaka, A., Kikuchi, K., *et al.* (2001) "Detection of antimitochondrial autoantibodies in immunofluorescent AMA-negative patients with primary biliary cirrhosis using recombinant autoantigens", *Hepatology* **34**, 243–248.

Palmer, J.M., Kirby, J.A. and Jones, D.E. (2002) "The immunology of primary biliary cirrhosis: the end of the beginning?", *Clin. Exp. Immunol.* **129**, 191–197.

Parikh-Patel, A., Gold, E., Mackay, I.R. and Gershwin, M.E. (1999) "The geoepidemiology of primary biliary cirrhosis: contrasts and comparisons with the spectrum of autoimmune diseases", *Clin. Immunol.* **91**, 206–218.

Parikh-Patel, A., Gold, E.B., Worman, H., Krivy, K.E. and Gershwin, M.E. (2001) "Risk factors for primary biliary cirrhosis in a cohort of patients from the united states", *Hepatology* **33**, 16–21.

Pena, S.D. and Chakraborty, R. (1994) "Paternity testing in the DNA era", *Trends Genet.* **10**, 204–209.

Prince, M.I., Chetwynd, A., Diggle, P., Jarner, M., Metcalf, J.V. and James, O.F. (2001) "The geographical distribution of primary biliary cirrhosis in a well-defined cohort", *Hepatology* **34**, 1083–1088.

Tanaka, A., Borchers, A.T., Ishibashi, H., Ansari, A.A., Keen, C.L. and Gershwin, M.E. (2001) "Genetic and familial considerations of primary biliary cirrhosis", *Am. J. Gastroenterol.* **96**, 8–15.

Triger, D.R. (1980) "Primary biliary cirrhosis: an epidemiological study", *Br. Med. J.* **281**, 772–775.

Tsuji, K., Watanabe, Y., van de Water, J., *et al.* (1999) "Familial primary biliary cirrhosis in Hiroshima", *J. Autoimmun.* **13**, 171–178.

Van de Water, J., Ishibashi, H., Coppel, R.L. and Gershwin, M.E. (2001) "Molecular mimicry and primary biliary cirrhosis: premises not promises", *Hepatology* **33**, 771–775.

Xu, L., Shen, Z., Guo, L., *et al.* (2003) "Does a betaretrovirus infection trigger primary biliary cirrhosis?", *Proc. Natl Acad. Sci. USA* (online before print June 27).

